# Spatio-temporal evaluation of the emergency capacity of the cross-regional rain-flood disaster in the Yangtze River Economic Belt in China

**DOI:** 10.1038/s41598-021-82347-5

**Published:** 2021-01-28

**Authors:** Qian Wang, Junfei Chen

**Affiliations:** 1 Business School, Hohai University, Nanjing, 210098 China; 2 Yangtze Institute for Conservation and Development, Hohai University, Nanjing, 210098 China; 3 Research Institute of Jiangsu Yangtze River Conservation and High-Quality Development, Nanjing, 210098 China

**Keywords:** Natural hazards, Environmental impact

## Abstract

This paper assesses the emergency capacity of rain-flood disaster in provinces along the Yangtze River Economic Belt (YREB), China, from 2013 to 2017. In this study, the evaluation index system of emergency capacity on rain-flood disaster was built from the economic-social and environmental-natural aspects, and spatial auto-correlation analysis was used to analyze spatial differentiation characteristics of the emergency capacity. Then, the Spatial Durbin Model (SDM) was used to analyze the influence mechanism of the development level of economic-social factors (ESF) subsystem, environmental-natural factors (ENF) subsystem and the coupling level of these two subsystems on the emergency capacity of rain-flood disaster in provinces. The findings show that the emergency capacity distribution of rain-flood disaster in the YREB presented a “decreasing” spatial pattern of eastern, central and western regions. The development of two subsystems has produced spatial spillover effect and diffusion effect on the neighboring areas. There was a high coupling degree between these two subsystems in the YREB. Although spillover effect existed in space, the spillover did not depend on economic distance.

## Introduction

The occurrence of rain-flood disaster often causes huge losses to local development. Therefore, rain-flood disaster has attracted attention from all walks of life. In recent years, the research hotspot about rain-flood disaster has focused on problems as follows: (1) what the causes of urban rain flood damage are^1,2^, (2) how to carry out loss assessment^3,4^ and risk assessment^5–9^, (3) how to use the rain flood resources effectively^10–14^, (4) how to solve the problem of non-point source pollution of rainwater and flood^15^, (5) what are the rain-flood management system characteristics of other countries^16,17^. As is known to all, the occurrence of rain-flood disaster often has characteristics of suddenness and complexity^18^, and efficient emergency capacity can effectively reduce the loss and negative impact of disaster. Therefore, the research on the emergency capacity of rain-flood disaster has practical significance. To date, studies on the emergency capacity of rain-flood disaster focuses on establishing models to simulate the process of disaster occurrence^19–21^, and provides the decision-making basis for urban rain-flood control. Most research works are limited to one region, there are few cross-regional studies. However, the urban planning and construction, the level of economic-social development and environment-natural development in the adjacent regions are mostly similar or even have spillover effects on each other. Therefore, it is necessary to study the emergency capacity of rain-flood disaster across regions. The Spatial Durbin Model (SDM) can be adapted for above problems. SDM is an analysis model for spatial effects, it can incorporate both independent variables and dependent variables into the model. By constructing spatial weight matrix and carrying out spatial regression analysis on relevant variables, SDM can explain the relationship and spillover effect between spatial variables better. At present, the SDM has been applied to environmental efficiency assessment^22^, green finance development^23^, medical insurance^24^ and other fields^25,26^. Therefore, this paper considered to use the model to study the emergency capacity of cross-regional rain-flood disaster.

In this paper, SDM was adopted to investigate the spillover effect of rain-flood disaster emergency capacity among provinces. Scientific basis for well-informed decision-making to improve the trans-provincial emergency capacity of rain-flood disaster from the perspective of regional coordinated development were also provided. The rest of this research is organized as follows. Section “Study area” introduces study area. Section “Data and methods” constructs the index system and evaluation model of the economic-social factors (ESF) subsystem and environmental-natural factors (ENF) subsystem. Section “Results and interpretation” takes 11 provinces of the YREB as the research object, and analyses the emergency capacity of rain-flood disaster in provinces under the interaction of two subsystems. Based on findings, Sect. “Conclusions and suggestions” provides conclusions and suggestions.

## Study area

**Figure 1:**
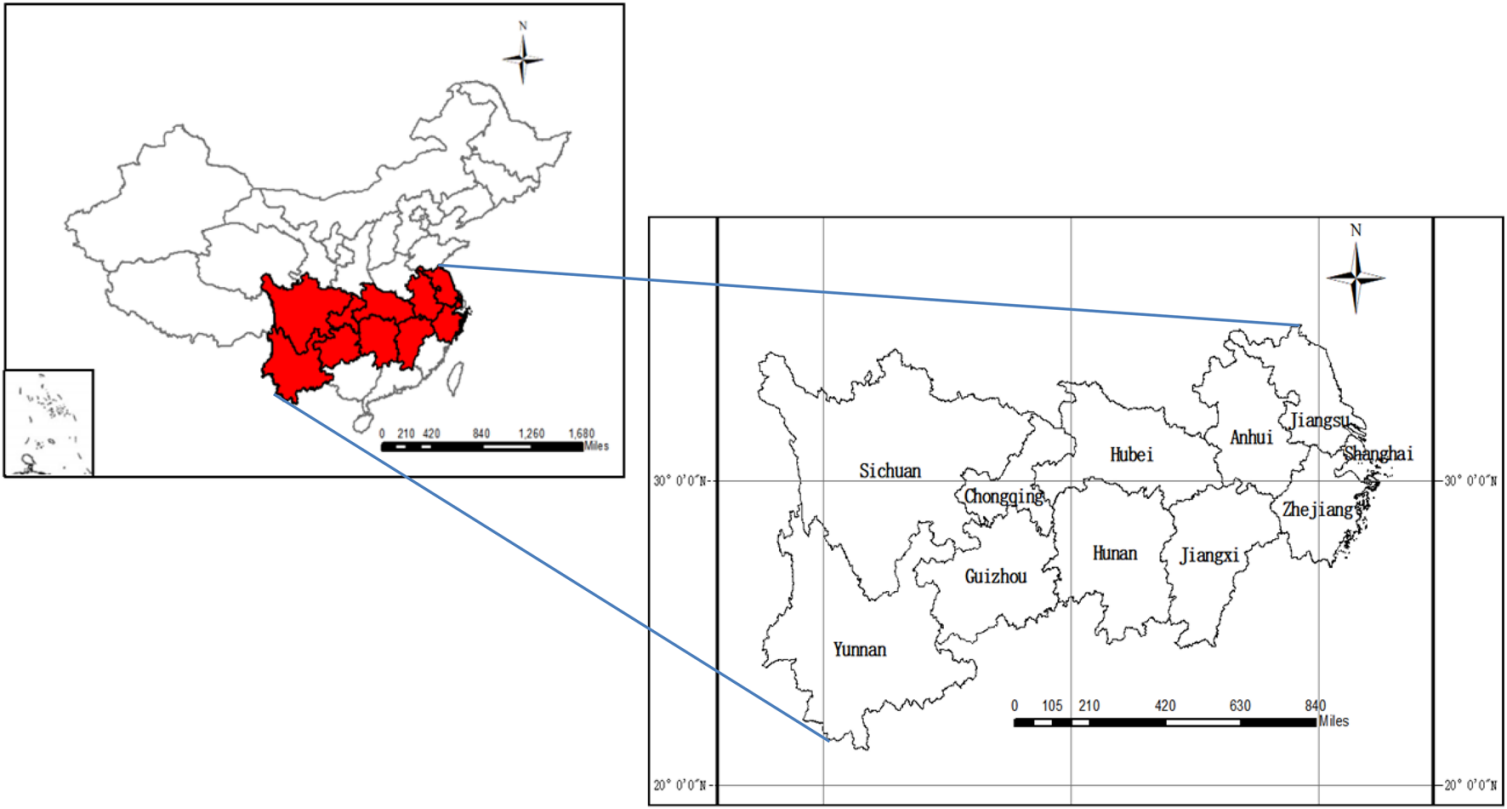
Location of the YREB, China. The map was generated by ArcGIS 10.5. https://www.esri.com/en-us/arcgis/products/districting-for-arcgis/overview.

Covering 11 provinces, including Shanghai, Jiangsu, Zhejiang, Anhui, Jiangxi, Hubei, Hunan, Chongqing, Sichuan, Guizhou and Yunnan, the Yangtze River Economic Belt(YREB) spans three major regions (i.e., the eastern, central and western regions of China) (Fig. 1). According to the statistical yearbook of China (2018), the YREB accounted for 42.9% of the population and 43.8% of the GDP in 2018. As one of the major national strategies, the YREB has become an engine of national development. However, most of the Yangtze River Basin belongs to the subtropical monsoon climate zone, where the rainfall is heavy and concentrated, and the rainy season lasts for a long time, leading to formation of flood superposition easily. In addition, with flat terrain and curved river channel, the flood discharge is poor in the middle reaches of the YREB. All of these lead to frequent rainstorms and floods in some provinces of the YREB, which seriously threaten the safety of people's lives and property, and cause great direct and indirect losses to agriculture, transportation and other industries as well. In late June 2017, heavy rains occurred in the Poyang Lake and Dongting Lake areas. Affected by the heavy rains, the No.1 flood occurred in the Yangtze River, affecting 16.4665 million people and causing a direct economic loss of 58.66 billion yuan. In mid-august 2018, heavy rainfall affected eight provinces including Shanghai, Jiangsu, Zhejiang, Anhui, etc., with 16.7764 million people affected and 32.385 billion yuan of direct economic loss. As one of the major disasters that seriously hamper the coordinated development of the YREB, the rain-flood disaster must be paid enough attention. Through the assessment of the emergency capacity of the rain-flood disaster and putting forward the management measures, this paper provided guidance for strengthening the rain-flood disaster management in the YREB.

## Data and methods

### Data source

Data in ESF subsystem, for example, GDP per capita, Proportion of secondary and tertiary industries, Urbanization level, Population density, etc., came from China Statistical Yearbooks. Proportion of expenditure on general public services to budgetary expenditure, Drainage pipe density in built-up area, Number of beds in hospitals and health centers per 10,000 people, Traffic density, Construction land density, etc., came from provincial statistical yearbooks. Public sector disaster response capacity, Public awareness of disaster relief are qualitative indicators, which were obtained by expert scoring method.

Date in ENF subsystem, for example, Proportion of cultivated land area, Forest coverage rate, Percentage of wetland area, Percentage of grassland area, Percentage of grassland area, etc., came from provincial statistical yearbooks, Annual precipitation came from flood and drought disaster bulletin.

### Construction of evaluation index system

Based on the existing studies^11,13–15,17,27^ and principle of systematization, rationality and availability, the evaluation index system is divided into two subsystems, i.e. the ESF subsystem and ENF subsystem (Table 1).

**Table 1:** Comprehensive evaluation index system.

Subsystem	Indicators	Attribute
*ESF* subsystem	GDP per capita (yuan)	+
	Proportion of secondary and tertiary industries (%)	+
	Urbanization level (%)	+
	Proportion of expenditure on general public services to budgetary expenditure (%)	+
	Population density (people/km^2^)	−
	Drainage pipe density in built-up area (km/km^2^)	+
	Number of beds in hospitals and health centers per 10,000 people (piece/10,000 people)	+
	Traffic density (km/km^2^)	+
	Construction land density (%)	−
	Public sector disaster response capacity	+
	Public awareness of disaster relief	+
*ENF* subsystem	Annual precipitation (mm)	−
	Proportion of cultivated land area (%)	+
	Forest coverage rate (%)	+
	Percentage of wetland area (%)	+
	Percentage of grassland area (%)	+
	Green coverage rate (%)	+

*ESF* economic-social factors, *ENF* environmental-natural factors.

The indicator of proportion of secondary and tertiary industries represents the proportion of output of secondary and tertiary industries in total output, the indicator of urbanization level represents the proportion of urban population in total population, public sector disaster response capacity and public awareness of disaster relief are qualitative indicators, which can be obtained by expert scoring method.

The ESF subsystem includes 11 indicators, such as per capita GDP, urbanization level, population density, etc., which reflect the respond ability of the provincial rain-flood disaster on the level of economic-social environment development. Indicators such as per capita GDP, urbanization level, the proportion of secondary and tertiary industries and proportion of expenditure on general public services to budgetary expenditure reflect the level of regional economic development and the characteristics of local industrial structure. Indicators such as population density, construction land density reflect the level of regional urbanization. Indicators such as drainage pipe density in built-up area, number of beds in hospitals and health centers per 10,000 people, and traffic density reflect the level of infrastructure construction and public facilities construction. Indicators such as public sector disaster response capacity and public awareness of disaster relief reflect the ability and awareness of local government and the public to cope with disasters. The ENF subsystem includes six indicators, such as average annual precipitation, proportion of cultivated land area, forest coverage rate, etc., which reflect the local natural conditions. The above indicators reflect the level of emergency capacity to rain-flood disaster on the level of environmental-natural development. In addition to the indicator of the annual precipitation, the higher the values of other indicators in the ENF subsystem, the stronger the emergency capacity of rain-flood disaster.

Considering the original data may affect the evaluation results due to the varied scales, the indicators were normalized according to the following method:

1 $$ x^{\prime}_{ij} = \left\{ {\begin{array}{*{20}l} {(x_{ij} - x_{ij\min } )/(x_{ij\max } - x_{ij\min } ),} \hfill & {{\text{When }}x_{ij} {\text{ is a positive indicator}}} \hfill \\ {(x_{ij\max } - x_{ij} )/(x_{ij\max } - x_{ij\min } )} \hfill & {{\text{When }}x_{ij} {\text{ is a negative indicator}}} \hfill \\ \end{array} } \right. $$

where, $x_{ij}$, $x_{ij\max }$, $x_{ij\min }$ represent the indicator *i* in time *j*, the upper limit and lower limit of $x_{ij}$, respectively.$x^{\prime}_{ij}$ represents the normalized value.

### Evaluation model of rain-flood disaster emergency capacity

The ESF subsystem of the provincial emergency capacity for rain-flood disaster contains indexes which are recorded as $X_{1} ,X_{2} ,...,X_{n}$. The ENF subsystem of the provincial emergency capacity for rain-flood disaster contains indexes which are recorded as $X^{\prime}_{1} ,X^{\prime}_{2} ,...,X^{\prime}_{m}$. The development level of these two subsystems can be expressed by the weighted synthesis of corresponding indexes:

2 $$ ES(t) = \sum\limits_{i = 1}^{n} {K_{i} (t)X_{i} (t)} ,\quad EN(t) = \sum\limits_{j = 1}^{m} {Z_{j} (t)X^{\prime}_{j} (t)} $$

where, $ES(t)$, $EN(t)$ represent the development index of economic-social factors and the development index of environmental-natural factors of the provincial rain-flood disaster emergency capacity during the period *t*, respectively, indicating the contribution of ESF subsystem and ENF subsystem to the rain flood emergency capacity, $K_{i} (t)$ and $Z_{j} (t)$, which are determined by factor analysis, represent weight coefficients of indicators in two subsystems.

The factor analysis method can determine indicators’ weights by constructing the factor analysis model according to the relationship between values of indicators. This method can dynamically assign the indicators’ weights with the change of environment, which is helpful to realize the dynamic monitoring of the evaluation object without losing objectivity. Therefore, it is widely applied in various studies^28,29^. In view of the wide applicability of factor analysis method, this method was used to determine the weight coefficients of indicators (i.e. $K_{i} (t)$ and $Z_{j} (t)$).

Considering that the coordination of ESF subsystem and ENF subsystem has an important impact on the emergency response capability of rain-flood disaster, the coupling index (CI) is defined to reflect the coordination degree of these two subsystems. The larger CI, the greater the degree of coordination between the two subsystems, and the stronger emergency capacity of the rain-flood disaster. Based on relevant studies^30,31^, CI is expressed mathematically as:

3 $$ CI = \sqrt {\frac{2ES(t) \times EN(t)}{{[ES(t)]^{2} + [EN(t)]^{2} }}} $$

In order to judge the emergency capacity of rain-flood disaster in varied provinces, the rain-flood disaster emergency capacity index (ECI) is defined. This index needs to embody not only the development level of ESF subsystem and ENF subsystem of rain-flood disaster emergency capacity, but also the impact of coordination between these two subsystems on the emergency capacity of rain-flood disaster. Therefore, it is defined as:

4 $$ ECI = \sqrt {TC \times CI} $$

where, TC represents the coordination coefficient of ESF subsystem and ENF subsystem synergy, $TC = a\;ES(t) + b\;EN(t)$, a and b are the undetermined weight coefficients, which are determined according to the importance of these two subsystems. TC reflects comprehensively the level of ESF subsystem and ENF subsystem development. The larger TC is, the higher the level of these two subsystems development of the province is, and the stronger the rain flood emergency capacity of the province is.

Referring to the segmentation standard of coupling degree from previous studies^32,33^ and the median segmentation method, the coupling degree of these two subsystems and emergency capacity level of the provincial rain-flood disaster are classified (Tables 2 and 3).

**Table 2:** Standard for coupling degree level.

CI	(0, 0.50]	(0.50, 0.75]	(0.75, 0.90]	(0.90, 1]
Degree	Weak coupling stage	Antagonistic stage	Running-in stage	Strong coupling stage

*CI* the coupling index.

**Table 3:** Classification standard for emergency capacity of rain-flood disaster.

ECI	(0, 0.50]	(0.50, 0.60]	(0.60, 0.70]	(0.70, 0.85]	(0.85, 1]
Degree	Very poor level	Poor level	Medium level	Good level	Excellent level

*ECI* emergency capacity index.

### Spatial econometric analysis

In order to study the spatial effect of ESF subsystem and ENF subsystem development level on the emergency capacity of the rain-flood disaster in provinces, this paper adopted spatial econometric analysis, which mainly involves autocorrelation test and spatial panel data model.

#### Spatial autocorrelation test

Global Moran's I index and local Moran's I index were used to analyze the spatial correlation of rain-flood disaster emergency capacity of varied provinces in the YREB.

In order to give fully consideration to the spatial geographical differences and economic differences among provinces, the following three weight matrices were introduced for analysis.

Geospatial adjacency weight matrix (*W*_*1*_):

5 $$ w_{ij} = \left\{ {\begin{array}{*{20}l} 1 \hfill & {When\;{\kern 1pt} region\;i\;and\;j\;have\;a\;common\;boundary} \hfill \\ 0 \hfill & {When\;region\;i\;and\;j\;have\;no\;common\;boundary;\;or\;i = j} \hfill \\ \end{array} } \right.{\kern 1pt} $$

Geographic distance weight matrix (*W*_*2*_):

6 $$ w_{ij} = \left\{ {\begin{array}{*{20}l} {{\raise0.7ex\hbox{$1$} \!\mathord{\left/ {\vphantom {1 {d^{2} }}}\right.\kern-\nulldelimiterspace} \!\lower0.7ex\hbox{${d^{2} }$}}} \hfill & {When\;i \ne j} \hfill \\ 0 \hfill & {When\;i = j} \hfill \\ \end{array} } \right. $$

Economic-geographic distance weight matrix (*W*_*3*_):

7 $$ w_{ij} = \left\{ {\begin{array}{*{20}l} {\frac{1}{{d^{2} }} \cdot \frac{1}{{\left| {\overline{P}_{i} - \overline{P}_{j} } \right|}}} \hfill & {When\;i \ne j} \hfill \\ 0 \hfill & {When\;i = j} \hfill \\ \end{array} } \right. $$

8 $$ \overline{P}_{i} = \sum\limits_{{t = T_{0} }}^{T} {{{P_{it} } \mathord{\left/ {\vphantom {{P_{it} } {(T - T_{0} + 1)}}} \right. \kern-\nulldelimiterspace} {(T - T_{0} + 1)}}} $$

where, *d* represents the Euclidean distance between region *i* and region *j*, $P_{it}$ represents the GDP per capita of the region *i* in year *t*, $\overline{P}_{i}$ and $\overline{P}_{j}$ represent the average GDP per year of region *i* and region *j*, respectively.

Global Moran's I index can be adopted to measure the global spatial autocorrelation:

9 $$ Moran^{\prime}s\;I = \frac{{\sum\limits_{i = 1}^{n} {\sum\limits_{j = 1}^{n} {w_{ij} (y_{i} - \overline{y})(y_{j} - \overline{y})} } }}{{S^{2} \sum\limits_{i = 1}^{n} {\sum\limits_{j = 1}^{n} {w_{ij} } } }} $$

where, $y_{i}$ and $y_{j}$ are the indicators of region *i* and region *j*, $\overline{y}$ is the mean value, $S^{2}$ is the variance, $w_{ij}$ is the weight matrix of region *i* and region *j*, and *n* is the number of regions. When Moran's I > 0, the index space is positively correlated. When Moran's I < 0, the index space is negatively correlated. When Moran's I = 0, the index space is irrelevant.

Since the Global Moran's I index can only judge the spatial clustering phenomenon of the whole research target, it cannot identify the spatial correlation pattern of the target. It is considered to use the local Moran's I index to further clarify the local spatial dependence of the rain flood emergency capacity of provinces in the YREB.

The essence of the local Moran's I index is to scatter the value of Global Moran's I index to each region. The calculation formula is as follows:

10 $$ I_{i} = \frac{{(x_{i} - \overline{x})}}{{s_{i} }}\sum\limits_{j = 1}^{n} {W_{ij} } (x_{j} - \overline{x}) $$

where, $x_{i}$, $x_{j}$ are the values of region *i* and region *j*, $\overline{x}$ is the mean value, $s_{i}$ is the variance, $W_{ij}$ is the weight matrix, and *n* is the number of regions.

#### Spatial Durbin model

The Spatial Durbin model (SDM) takes both the lag effect and the error effect into account, and considers the effects of the lag explained variable and the lag explanatory variables on the explained variable as well^25,26,34^. Its general form is:

11 $$ y = \rho Wy + \beta X + WX\theta + \mu + \varepsilon $$

where, *y* represents the explained variable, *X* represents the explanatory variables (or control variables), *W* represents the spatial weight matrix, $\beta$, $\theta$ are parameters, $\mu$ represents control regional effects, $\varepsilon$ is the residual term.

According to the above model, variables $G(t)$, $ES(t)$, $EN(t)$, $CI(t)$ and $ECI(t)$ are introduced to establish the following model:

12 $$ \begin{aligned} \ln ECI_{i} (t) & = C_{i} (t) + \rho W\ln ECI_{i} (t) + \beta_{1} \ln G_{i} (t) + \beta_{2} \ln ES_{i} (t) + \beta_{3} \ln EN_{i} (t) + \beta_{4} \ln CI_{i} (t) \\ & \quad + \theta_{{1}} W\ln G_{i} (t) + \theta_{2} W\ln ES_{i} (t) + \theta_{3} W\ln EN_{i} (t) + \theta_{4} W\ln CI_{i} (t) + \mu_{i} + \varepsilon_{i} \\ \end{aligned} $$

where, $C_{i}$ is a constant, $W$ is the spatial weight matrix, $\varepsilon_{i}$ is the random error term, $\mu_{i}$ is the regional effect. $G_{i} (t)$ is the relative index of provincial GDP, which taking the impact of the comprehensive development level of provinces into account. It is expressed as:

13 $$ G_{i} (t) = {{AGDP_{it} } \mathord{\left/ {\vphantom {{AGDP_{it} } {AGDP_{0t} }}} \right. \kern-\nulldelimiterspace} {AGDP_{0t} }} $$

where, $AGDP_{it}$ is the per capita GDP of the region *i* in year *t*, and $AGDP_{0t}$ is the national per capita GDP in year *t*. Since the effective emergency capacity of rain-flood disaster in the province is affected by the per capita GDP of the province in the previous year. The data of the previous year shall be used for correction when evaluating the emergency capacity of rain-flood disaster in the actual analysis.

## Results and interpretation

### Analysis of rain-flood disaster emergency capacity

After the 18th National Congress of the Communist Party of China in November 2012, the concept of the Yangtze River Economic Belt was formed and upgraded to the national strategy. The cross-regional cooperation mechanisms have gradually formed in the Yangtze River Basin since then. And some data after 2018 is still unavailable. Therefore, based on the above evaluation index system, the data of 11 provinces in the YREB from 2013 to 2017 was analyzed, and the weight of each index in the ESF subsystem and ENF subsystem was calculated by factor analysis method. The panel data composed of three provincial indexes was obtained, i.e. the development index of economic-social factors (ES), the development index of environmental-natural factors (EN), the coupling index (CI), as shown in Table 4.

**Table 4:** Provincial index calculation results.

Province	ES					EN					CI				
	2013	2014	2015	2016	2017	2013	2014	2015	2016	2017	2013	2014	2015	2016	2017
Shanghai	0.84	0.59	0.82	0.80	0.87	0.40	0.58	0.57	0.34	0.45	0.88	1.00	0.97	0.85	0.90
Jiangsu	0.67	0.70	0.63	0.73	0.68	0.65	0.70	0.69	0.68	0.44	1.00	1.00	1.00	1.00	0.95
Zhejiang	0.55	0.64	0.62	0.69	0.72	0.55	0.42	0.37	0.43	0.38	1.00	0.96	0.94	0.95	0.91
Anhui	0.28	0.43	0.32	0.50	0.42	0.61	0.57	0.60	0.62	0.35	0.87	0.98	0.91	0.99	0.99
Jiangxi	0.27	0.43	0.29	0.40	0.35	0.57	0.44	0.51	0.56	0.63	0.88	1.00	0.93	0.97	0.92
Hubei	0.45	0.60	0.52	0.51	0.49	0.49	0.45	0.35	0.39	0.37	1.00	0.98	0.96	0.98	0.98
Hunan	0.30	0.53	0.40	0.34	0.34	0.44	0.36	0.38	0.43	0.37	0.96	0.96	1.00	0.99	1.00
Chongqing	0.50	0.60	0.52	0.57	0.61	0.62	0.44	0.45	0.55	0.3	0.99	0.98	0.99	1.00	0.94
Sichuan	0.31	0.40	0.34	0.40	0.40	0.34	0.16	0.29	0.43	0.53	1.00	0.82	0.99	1.00	0.98
Yunnan	0.14	0.32	0.18	0.24	0.13	0.49	0.41	0.24	0.37	0.46	0.73	0.99	0.98	0.96	0.72
Guizhou	0.25	0.46	0.24	0.33	0.28	0.39	0.25	0.22	0.34	0.29	0.96	0.92	1.00	1.00	1.00

*ES* the development index of economic-social factors, *EN* the development index of economic-social factors, *CI* the coupling index.

As can be seen from Table 4, the coupling index (CI) is above 0.9 in most provinces, it indicates that the development level of economic-social environment and environmental-natural in most provinces is in strong coupling state. Because the provinces have gradually realized the importance of ecological environment construction, while promoting economic development.

ECI in 11 provinces of the YREB from2013 to 2017 can be obtained according to Table 4.

**Figure 2:**
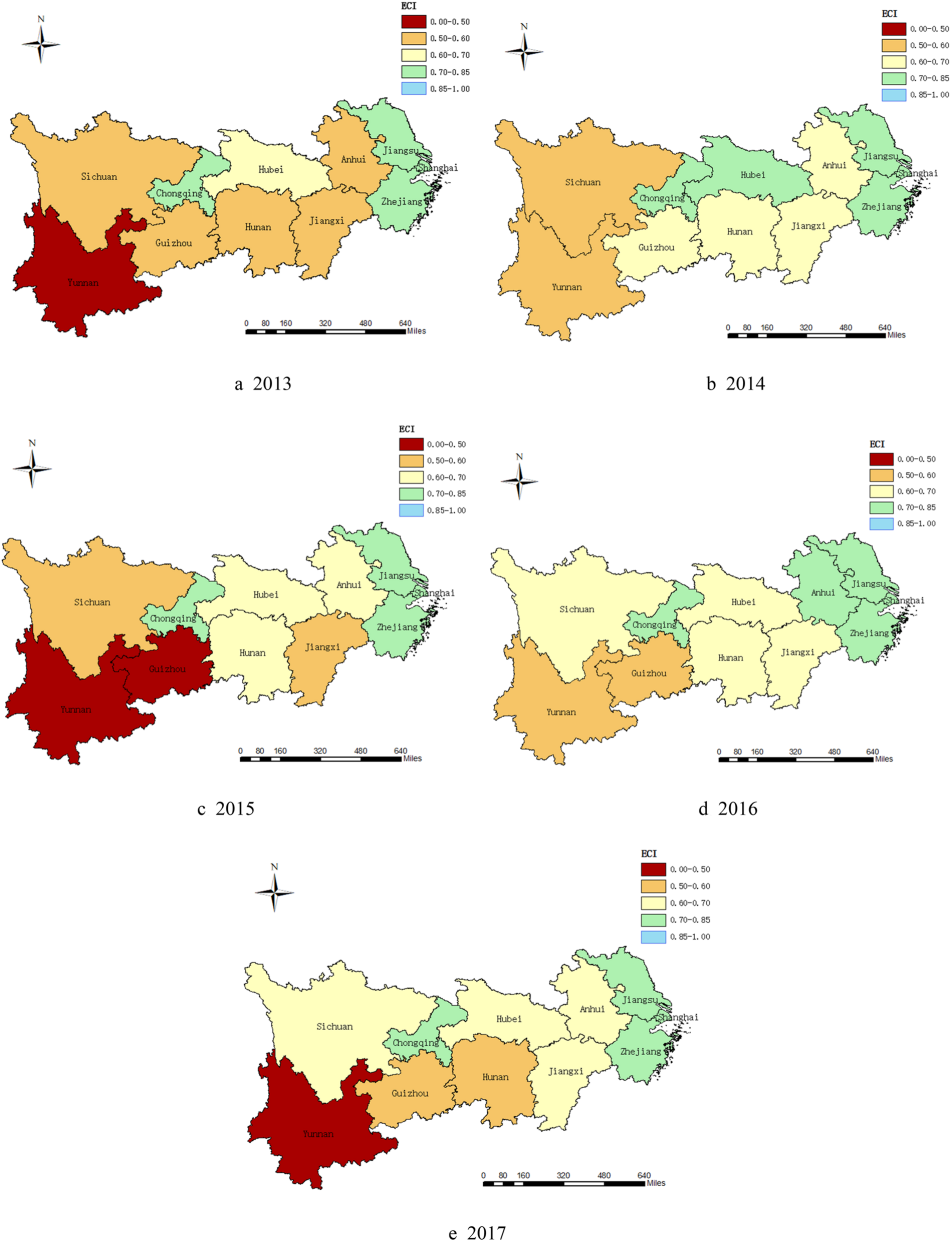
Emergency capacity level of flood disaster in the YREB during 2013–2017. (**a**) Level of regional ECI in 2013, (**b**) level of regional ECI in 2014, (**c**) level of regional ECI in 2015, (**d**) level of regional ECI in 2016, (**e**) level of regional ECI in 2017. The maps were generated by ArcGIS 10.5. https://www.esri.com/en-us/arcgis/products/districting-for-arcgis/overview.

As shown in Fig. 2, the emergency capacity level of flood disaster varies significantly among different provinces. Shanghai, Jiangsu and Zhejiang provinces have formed strong emergency capacity for rain-flood disaster from 2013 to 2017. On the one hand, it benefited from high social development level and strong economic strength. With sound economic foundation and rich public service resources as backing, these provinces can respond quickly, and reduce the loss and negative impact caused by disasters. On the other hand, compared with the middle and upper reaches of the YREB, these provinces have formed better eco-environmental protection and governance mechanisms, which produce a defense mechanism against rain-flood disaster. The emergency capacity of Anhui, Jiangxi, Hubei and Sichuan was mostly at a medium level over these years, because these provinces were positioned as grain production base and energy raw material production base in the national productivity layout, which had an impact on local economic-social development to some extent, especially in urban infrastructure, medical level, communication level and other aspects. When the rain-flood disaster occurred, the emergency response and processing capacity of the disaster were restricted. Chongqing had a strong emergency capacity of rain-flood disaster from 2013 to 2017, but the emergency capacity level of neighboring provinces such as Hunan, Guizhou, Yunnan is not ideal. Because in the development process of Chongqing, high-quality resources such as talents, technology and funds were absorbed from the surrounding provinces, leading to the results of the surrounding provincial economy weakening, the gap of economic-social development and the rain-flood disaster emergency capacity widening. To a certain extent, the emergency capacity can be reflected through the economic losses caused by rain-flood disaster^35^. According to the data of China Statistical Yearbook and China Flood and Drought Disaster Bulletin in 2017, the proportion of losses caused by rain-flood disaster in Shanghai, Jiangsu, Zhejiang, Chongqing was less than 0.1% of the total output value. The proportion of losses in areas such as Anhui, Jiangxi, Hubei, Sichuan was between 0.1 and 0.3%, while the proportion of losses in Guizhou, Yunnan and Hunan was generally more than 0.3%. Therefore, the above analysis results were basically consistent with the actual situation.

In conclusion, from 2013 to 2017, the coupling level of *ESF* subsystem and *ENF* subsystem was not consistent with the emergency capacity level of rain-flood disaster. In most provinces, these two subsystems were in a strong coupling stage, while the distribution of the emergency capacity formed a “decreasing” spatial pattern from the east to the west.

### Spatial econometric analysis

#### Spatial correlation analysis

As can be seen from Table 5, the Moran's I over these years passed the test at a significance level of 1%, indicating that the emergency capacity distribution of rain-flood disaster in various provinces of the YREB had an obvious spatial correlation.

**Table 5:** Moran's I index, from 2013 to 2017.

*t*	2013	2014	2015	2016	2017
$I$	0.366	0.439	0.494	0.473	0.377
$Z$	2.313	2.775	3.022	2.914	2.587
$p$	0.010	0.003	0.001	0.002	0.005

Confidence level: 0.99.

**Figure 3:**
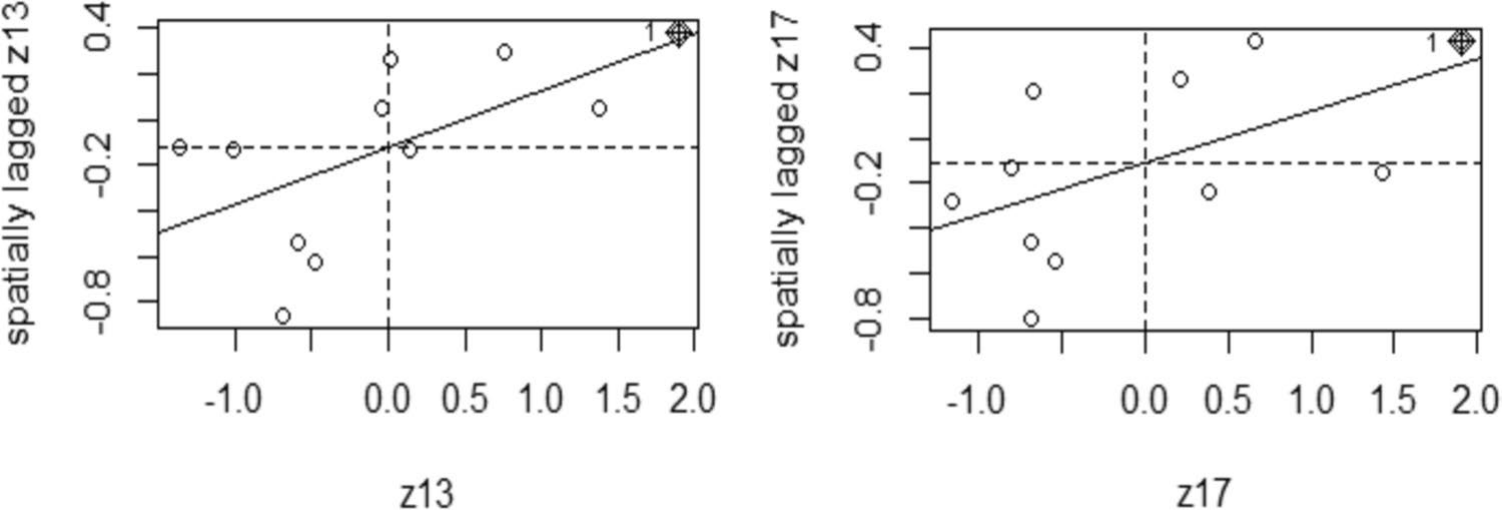
Local Moran scatter diagram in 2013 and 2017. It represents a form of spatial connection surrounded by regions. Moran scatter diagram consists of four quadrants. The points in the first and third quadrants (i.e. the upper right one and the lower left one) indicate that high values are surrounded by high values and low values are surrounded by low values, respectively, which means that areas with similar attributes are clustered together. The points in the second and fourth quadrants (i.e. the upper left one and the lower right one) indicate that high values are surrounded by low values or low values are surrounded by high values, which means that areas with different attributes are clustered together^36^. *z13* local Moran's I index in 2013, *z17* local Moran's I index in 2017.

Figure 3 shows the Moran scatter diagram of 2013 and 2017, indicating the local spatial agglomeration characteristics of rain-flood disaster emergency capacity of provinces.

As can be seen from Fig. 3, most of the points are located in the first and third quadrants, indicating that the emergency capacity of rain-flood disaster in provinces presents a positive spatial correlation. In 2017, eight provinces fell into the first and third quadrant, including Shanghai, Jiangsu, Zhejiang, Anhui, Jiangxi, Hubei, Hunan, Chongqing, indicating that it has formed the distribution of strong gathering in space. Sichuan, Guizhou and Yunnan fell into the second and fourth quadrant, indicating that the emergency capacity of rain-flood disaster in these provinces is unbalanced in spatial distribution. By comparing the spatial agglomeration state in 2013 and 2017, there was slightly increasing in the number of high-agglomeration provinces and low-agglomeration provinces.

Based on the spatial autocorrelation analysis of the rain-flood disaster emergency capacity of 11 provinces in the YREB, it can be preliminarily concluded that the rain-flood disaster emergency capacity of all provinces has significant characteristics of regional dependence, therefore the spatial econometric model is considered for analysis.

#### Analysis of econometric model results

Based on the indicators of ES, EN, CI, ECI and relevant data of 11 provinces along the YREB from 2013 to 2017, the econometric model of emergency capacity was built for quantitative analysis. The Wald test indicated the value passed the test of significance. Therefore, the spatial Durbin model (SDM) was used for modeling.

Tables 6 and 7 show the model estimation and effect decomposition results.

**Table 6:** The SDM model estimation results.

Indicators	Geographic adjacency matrix		Geographic distance matrix		Economic geographic distance matrix	
	Parameter	p value	Parameter	p value	Parameter	p value
ln *ECI*	− 0.757	4.1e − 06***	− 0.286	0.058	− 0.275	0.035
ln *G*	0.039	0.202	0.047	0.142	0.040	0.478
$\ln ES$	0.281	2.2e − 16***	0.273	2.2e − 16***	0.270	2.2e − 16***
$\ln EN$	0.148	1.019e − 11***	0.144	1.292e − 08***	0.120	1.614e − 06***
$\ln CI$	0.359	4.176e − 05***	0.364	0.0003056***	0.397	1.646e − 05***
$W * \ln G$	− 0.070	0.245	− 0.113	0.158	− 0.078	0.268
$W * \ln ES$	0.306	2.410e − 05***	0.242	0.0127399*	0.178	0.001**
$W * \ln EN$	0.148	0.001508**	0.066	0.267	0.096	0.02567*
$W*\ln CI$	0.443	0.004820**	0.387	0.102	0.208	0.147

*, **and*** mean significant at the confidence level of 10%, 5% and 1%, respectively.

**Table 7:** Spatial spillover effect of emergency capacity.

Weight matrix	Effect	ln ES	ln EN	ln CI
Geographic adjacency matrix	Direct effect	0.327	0.172	0.417
	Indirect effect	− 0.167	− 0.088	− 0.213
	Total effect	0.160	0.084	0.204
Geographic distance matrix	Direct effect	0.279	0.147	0.371
	Indirect effect	− 0.067	− 0.035	− 0.089
	Total effect	0.212	0.112	0.282
Economic geographic distance matrix	Direct effect	0.279	0.124	0.410
	Indirect effect	− 0.067	− 0.030	− 0.099
	Total effect	0.212	0.094	0.311

The direct effect, indirect effect and total effect can be obtained by decomposing the spatial spillover effect of the parameters listed in SDM model. Among them, direct effect refers to the influence brought by local parameters, while indirect effect refers to the influence brought by parameters from adjacent areas. The total effect is the sum of the two effect values above^37^.

Confidence level: 0.95.

As can be seen from Table 6, explanatory variables have significant impact on the emergency capacity of rain-flood disaster. Firstly, the elasticity coefficients of lnES and lnEN are positive, indicating that the level of economic-social development and the environmental-natural improvement are conducive to improving the emergency capacity of rain-flood disaster. The coupling index (CI) have positive effects on the emergency capacity of rain-flood disaster. It shows that the level of the two subsystems coordinated development has an important impact on the emergency capacity of rain-flood disaster in provinces. Secondly, the three indexes show positive spillover effects in space. It suggests that the impact of subsystems on neighboring provinces has positive externality. This is mainly because the spontaneous collaborative construction increases the opportunity of provincial interaction, and promotes the emergency capacity of rain-flood disaster in the province and adjacent areas. Thirdly, considering and comparing the analysis of geographical adjacency factors and additional economic geographical distance factors, the coefficient of lnCI is significant under the pure geographical adjacency factors, indicating that geographical location factors have certain externalities. However, the coefficients of lnCI are no longer significant after adding the geographical distance factors and economic distance factors. This may be due to the problems in the flow and allocation mechanism of various elements between provinces, when the provincial space distance is larger or economic gap is larger, the overflow would be restricted.

As can be seen from Table 7, the spatial direct effect, indirect effect and total effect of each variable are calculated. The specific analysis is as follows. Firstly, the direct effects of lnES and lnEN are positive, reflecting that the agglomeration of economic-social factors and environmental-natural factors in provinces has a positive impact on the emergency capacity of rain-flood disaster. Secondly, the indirect effect of both is negative. Because the unbalanced development of the society level makes adjacent provincial gap. The relatively backward areas, whose high-quality production factors are attracted by the relatively developed areas around, are difficult to live up to their potential. But at the same time, these areas have to undertake a huge task of ecological environment construction, which would undoubtedly produce negative influence on the improvement of rain-flood disaster emergency capacity. Thirdly, the direct effect of lnCI is positive and the indirect effect is negative. It shows that the coordinated development of these two subsystems produces demonstration effect and driving effect on the improvement of the emergency capacity. At the same time, no positive spillover effect except a weak "siphon effect" is produced on the surrounding provinces. No matter under the condition of geographical factors or economic factors, the total effects of lnCI are always positive. It indicates that level of the ESF subsystem and ENF subsystem coordination always has a great impact on provincial rain-flood disaster emergency capacity and produces positive spillover effects. Therefore, it is imperative to enhance the coordination of the provincial sub-systems and promote the harmonious development of economic society and natural environment.

## Conclusions and suggestions

### Conclusions and discussion

Based on the above research, the following conclusions can be drawn. First, the distribution of rain-flood disaster emergency capacity level in all provinces along the YREB presented a stepped distribution from east to west, which had characteristics of concentration and heterogeneity in time and space. To be specific, provinces in the lower reaches of the YREB, such as Shanghai, Jiangsu, Zhejiang, had relatively strong emergency capacity of rain-flood disaster, while provinces in the upper reaches, such as Guizhou and Yunnan, had relatively weak emergency capacity of rain-flood disaster. Second, according to the analysis results of the SDM, the elasticity coefficients of lnES and lnEN were significantly different from zero, indicating that the development of ESF subsystem and ENF subsystem has generated spatial spillover effect and diffusion effect on the neighboring areas. In addition, there was a high coupling degree between ESF subsystem and ENF subsystem in the YREB, with the values of CI above 0.9 in most provinces. According to the analysis results of three weight matrices (i.e. geospatial adjacency weight matrix, geographic distance weight matrix and economic-geographic distance weight matrix), the coefficients of lnCI were no longer significant after adding the geographical distance factors and economic distance factors, indicating that spillover effect of emergency capacity existed in space, it did not depend on economic distance. Therefore, when the provincial space distance was larger or economic gap was larger, the overflow would be restricted.

**Figure 4:**
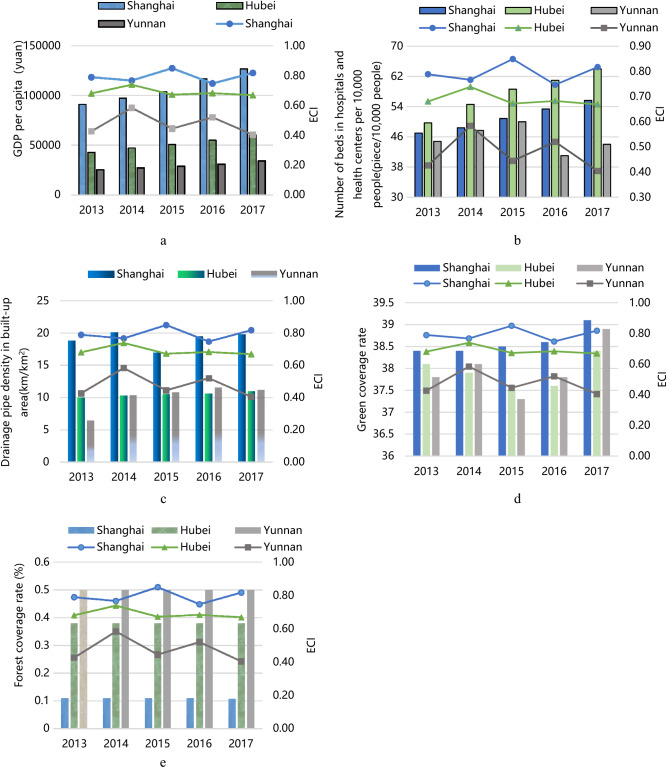
The influence of important indicators on the emergency response ability of representative cities. Bar charts display the important indicators (i.e. GDP per capita, number of beds in hospitals and health centers per 10,000 people, drainage pipe density in built-up area, green coverage rate and forest coverage rate) in Shanghai, Hubei, Yunnan. Line charts display the ECI in Shanghai, Hubei, Yunnan.

The above conclusions were based on the analysis of ESF subsystem and ENF subsystem. In order to further analyze the impact of relatively important indicators in these two subsystems on the emergency capacity of rain-flood disaster in the YREB, this paper selected five representative indicators (i.e. GDP per capita, number of beds in hospitals and health centers per 10,000 people, drainage pipe density in built-up area, green coverage rate, forest coverage rate) with high weight (above 0.1) in the evaluation index system for discussion. From the previous analysis, it can be seen that the emergency capacity of adjacent areas had the characteristics of spatial agglomeration. Therefore, this paper took Shanghai, Hubei and Yunnan as representative areas to analyze the spatio-temporal impact of above five indicators on the emergency capacity of rain-flood disaster, as shown in Fig. 4.

As can be seen from Fig. 4, from 2013 to 2017, in addition to the indicator of forest coverage rate, other four indicators of Shanghai were at high level, and showed a steady growth trend, the ECI of Shanghai was always ahead of Hubei and Yunnan. It can be inferred that indicators such as GDP per capita, number of beds in hospitals and health centers per 10,000 people, drainage pipe density in built-up area, green coverage rate had a positive impact on emergency capacity of rain-flood disaster. From 2013 to 2017, in addition to the high level of green coverage rate and forest coverage rate, the level of other three indicators of Yunnan was lower than those of Shanghai and Sichuan, and the emergency capacity of Yunnan was also lower than that of Shanghai and Sichuan. It indicated that the influence of economic-social factors were likely to be greater than that of environmental-natural factors. This may be because in recent years, the government and society have paid more attention to economic and social development, and made great efforts in these aspects, leading to the economic-social factors were more likely to change compared with the environmental-natural factors. Therefore, the influence of economic-social factors on the regional emergency capacity of rain-flood disaster became more obvious. According to the data of China Flood and Drought Disaster Bulletin released by the Ministry of Water Resources in 2018, the scopes of cumulative rainfall during the flood season in Shanghai, Hubei, Yunnan were 600–800 mm, 600–800 mm, 800–1000 mm, respectively. There was not much difference in scopes of cumulative rainfall among the three areas. But losses caused by rain-flood disasters reached 84 million yuan in Shanghai, 1.9 billion yuan in Hubei and 6.1billion yuan in Yunnan in 2018. It can be inferred that, although the disaster has characteristics of suddenness and unpredictable^18^, the difference in emergency capacity of rain flood caused by economic-social factors are one of the reasons for the huge loss gap^20^.

### Countermeasures and suggestions

First, the local government should optimize public service resources and narrow the gap in public service facilities along the YREB. The difference in economic development level of provinces in the YREB leads to the gap in public service resources, such as public medical resources and information service resources, and then lead to the gap in the emergency capacity of rain-flood disaster among provinces. As a consequence, it can be considered to strengthen the construction of relevant provincial medical institutions, improve the overall medical level of urban agglomerations, encourage the cross-provincial and cross-regional construction of large medical and health institutions to narrow the gap in the emergency capacity of provincial rain-flood disaster.

Second, the level of coordination and integration of infrastructure should be improved in the YREB. The imperfection of infrastructure will reduce the efficiency of economic and social development, leading to the gap in the emergency capacity of rain-flood disaster among provinces. Therefore, it is urgent to strengthen the provincial infrastructure integration construction. On the one hand, the government needs to optimize the layout of the road and rail networks, and form inter-city transportation networks among urban agglomerations and provinces to support the efficiency of inter-city transportation. Based on these, urban agglomerations can help each other and coordinate management timely when disasters occurs. On the other hand, the existing drainage pipeline networks should be reconstructed and expanded reasonably to improve the underground drainage and water storage capacity of urban agglomerations.

Third, regional flood control pattern should be built and coordinated construction of ecological environment should be strengthened. After the long-term development and destruction of human beings, the ecosystem of Yangtze river basin has gradually degenerated, and the threat of rain-flood disaster always exists. All provinces of the YREB cannot be immune from this situation. The ecological environment of some provinces will have an impact on the emergency capacity of rain-flood disaster in their own provinces as well as neighboring provinces. Therefore, the method of partition management is infeasible. Provinces of the YREB should have the awareness of regional flood control pattern, by controlling some key regions and spatial locations and establishing the flood detention system in these basins, strive to reduce the rain flood damage losses as much as possible. At the same time, various provinces should try to build the ecological environment in coordination, such as the construction of forest system, cross regional ecological compensation system, etc., to achieve the purpose of sharing resources and achieving coordinated development.

## Data Availability

The data reported in this paper are available and archived on the website of the National Bureau of Statistic (http://www.stats.gov.cn/tjsj/ndsj/) and the Ministry of Water Resources of the People’s Republic of China (http://www.mwr.gov.cn/sj/tjgb/zgshzhgb/).

## References

[1] Yin J, Ye MW, Yin ZE, Xu SY (2015). A review of advances in urban flood risk analysis overChina. Stoch. Environ. Res. Risk A..

[2] Tran D, Xu DW, Dang V, Alwah AAQ (2020). Predicting urban waterlogging risks by regression models and internet open-data sources. Water.

[3] Lan JT (2013). Research on the evaluation index system of flood disaster loss. China Popul. Resour. Environ..

[4] Zhou L, Wu XH, Ji ZH (2017). Research advance in flood damage assessment considering resilience. J. Nat. Disasters..

[5] Lamb R (2010). A new method to assess the risk of local and widespread flooding on rivers and coasts. J. Flood Risk Manag..

[6] Zhang Q, Zhang JQ, Jiang LP, Liu XP, Tong ZJ (2014). Flood disaster risk assessment of rural housings: A case study of Kouqian Town in China. Int. J. Environ. Res. Pu..

[7] Quan RS (2014). Risk assessment of flood disaster in Shanghai based on spatial-temporal characteristics analysis from 251 to 2000. Environ. Earth Sci..

[8] Towe R, Tawn J, Lamb R, Sherlock C, Liu Y (2016). Improving statistical models for flood riskassessment. Flood Risk..

[9] Chen LL, Zhang XD, He F, Yuan RS (2019). Regional green development level and its spatial relationship under the constraints of haze in China. J. Clean Prod..

[10] Chen YR, Yeh CH, Yu BF (2011). Integrated application of the analytic hierarchy process and the geographic information system for flood risk assessment and flood plain management in Taiwan. Nat. Hazards..

[11] Chen JF, Wang HM (2012). Flood and Drought Disaster Risk Management Theory and Application.

[12] Wang HM, Liu GF, Tong JP, Qiu L (2012). Study on dynamic emergency decision-making mode of unconventional water disaster. Soft Science..

[13] Jungwon Y, Louise KC (2017). An expected event, but unprecedented damage: Structure and gaps of large-scale response coordination of the 2011 Thailand floods. Disaster Prevent. Manag..

[14] Li P, Sheng MY, Yang DW, Tang LH (2019). Evaluating flood regulation ecosystem services under climate, vegetation and reservoir influences. Ecol. Indic..

[15] Kuoppamaki K, Hagner M, Lehvavirta S, Setala H (2016). Biochar amendment in the green roof substrate affects runoff quality and quantity. Ecol. Eng..

[16] Pan WF, Ke JY, Zheng P, Zhan X (2018). Flood control effects of low-impact development on urban water logging node under different rainfall characteristics. China Environ. Sci..

[17] Brillinger M, Dehnharst A, Schwarze R, Albert C (2020). Exploring the uptake of nature-based measures in flood risk management: Evidence from German federal states. Environ. Sci. Policy..

[18] Wei YM, Jin JL, Zhou CH, Wan Q, Li JR (1997). A study on the system of flood disaster estimation. J. Catastrophol..

[19] Bates PD, De Roo APJ (2000). A simple raster-based model for flood inundation simulation. J. Hydrol..

[20] Yin J (2017). Urban pluvial flood scenario modeling and emergency response assessment using high resolution Lidar-DSM. Geogr. Res..

[21] Zheng P, Wang BL, Chen ZJ, Pan WF, Huang JH (2019). Urban stormwater and flood control effect of green and grey infrastructures under extreme rainfall conditions. China Environ. Sci..

[22] Chen JF, Chen MC, Gao SP, Xu JP, Zhou P (2019). Risk assessment of rain flood disaster in Nanjing city based on cloud matter-element model. J. Econ. Water Resour..

[23] Ren M, Huang CH, Wang XM, Hu W, Zhang WX (2019). Research on the distribution of Pollution-Intensive industries and their spatial effects in China. Sustainability..

[24] Jiang LT (2019). A research on the relationship between public medical insurance and private health insurance in China—Analysis based on spatial durbin model. J. Financial Dev. Res..

[25] Lapple D, Kelley H (2015). Spatial dependence in the adoption of organic drystock farming in Ireland. Eur. Rev. Agric. Econ..

[26] Tian W, Jia JQ, Bo N, Yin LS (2018). A research on the spatial effect of inter provincial income gap in china —an empirical analysis based on the SDM model. East China Econ. Manag..

[27] Li XS, Long XX, Qi XX (2019). Dynamic evolution and analysis of coupling development of economy, society and environment in Yangtze River Economic Belt. Resour. Environ. Yangtza Basin..

[28] Randjelovic D, Stankovic J, Jankovic-Milic V, Stankovic J (2013). Weight coefficents determination based on parameters in factor analysis. Metal. Int..

[29] Meng XL, Xing MY (2019). Research on the Hubei high-quality development comprehensive evaluation under the background of supply side structural reform based on the weighted factor analysis. J. Appl. Stat. Manag..

[30] Tao CQ (2016). Frontier Theory and Application of Spatial Econometrics.

[31] Chen JF, Yu XY, Qiu L, Deng MH, Dong R (2018). Study on vulnerability and coordination of Water-Energy-Food System in northwest China. Sustainability..

[32] Li Q, Wei W (2019). Study on the coupling coordination degree between economic growth quality and ecological environment optimization in the Yangtze River Economic Belt. Soft Sci..

[33] Zhou ZZ, Wang JL (2019). Research on the coupling coordinated development of ecological environment pressure, state and response in Yangtze River Economic Belt. Sci. Technol. Manag. Res..

[34] Shahnazi R, Shabani ZD (2019). The effects of spatial spillover information and communications technology on carbon dioxide emissions in Iran. Environ. Sci. Pollut. Res..

[35] Li N, Zhang ZT, Chen X, Feng JL (2017). Importance of economic loss evaluation in natural hazard and disaster research. Progress Geogr..

[36] Zhu DY, Sun RY (2019). Analysis on spatial allocation of vertical transfer payment-based on dynamic spatial durbin model. Tax Econ. Res..

[37] Hui ECM, Lian GC (2016). Spatial spillover effect of urban landscape views on property price. Appl. Geogr..

